# Screening for Obstructive Sleep Apnea in Type 2 Diabetes Patients – Questionnaires Are Not Good Enough

**DOI:** 10.3389/fendo.2016.00124

**Published:** 2016-09-13

**Authors:** Katerina Westlake, Jan Polak

**Affiliations:** ^1^2nd Internal Medicine Department, Vinohrady Teaching Hospital, Prague, Czech Republic; ^2^Third Faculty of Medicine, Center for Research on Diabetes, Metabolism and Nutrition, Charles University in Prague, Prague, Czech Republic

**Keywords:** type 2 diabetes, cardiovascular risk factor, sleep apnea, screening, Berlin questionnaire, STOP Bang questionnaire, home sleep monitor

The shorter life expectancy of patients with type 2 diabetes caused by cardiovascular and cerebrovascular diseases compels clinicians to make a systematic effort to address traditional cardiovascular risk factors, such as dyslipidemia, hypertension, hyperglycemia, obesity, and smoking. However, another major cardiovascular risk factor substantially influencing the quality and length of life – obstructive sleep apnea syndrome (OSA) – is frequently overlooked. OSA is characterized by a repetitive upper airway obstruction during sleep leading to intermittent hypoxemia and sleep fragmentation. Numerous epidemiological studies have proved that untreated OSA represents an independent risk factor significantly increasing all cause as well as cardiovascular, cerebrovascular, and cancer mortality (1–7). The importance of OSA is underscored by its considerable prevalence ranging from 5 to 10% in the general population and is even higher in the population of type 2 diabetes patients where a prevalence of ~70% was reported (8–10). Furthermore, ~90% of all subjects suffering from moderate or severe OSA remain undiagnosed (11).

Acknowledging the clinical significance of untreated OSA, the American Diabetes Association’s Standards of Care recommend the active treatment of OSA when diagnosed (12), while the International Diabetes Federation’s guidelines emphasize the need for systematic OSA screening in all patients with type 2 diabetes (13). The suggested screening approach is split into two steps. In step one, patients fill in a questionnaire stratifying respondents into high or low risk of having OSA. Subsequently, in step two, high-risk patients undergo home sleep monitoring consisting of overnight registration of pulse oxymetry, preferably combined with nasal flow measurement and other accessory signals. Although such a two-step approach seems intuitively attractive, it should be noted that there are currently no studies supporting this approach or validating any of the available questionnaires in a population of type 2 diabetes patients. In fact, the recommendation suggesting a two-step OSA screening procedure is not based on any scientific evidence. Since the performance characteristics of screening questionnaires substantially influence the outcome of the whole screening procedure, this article scrutinizes the key features of available questionnaires and their performance. Subsequently, we will discuss the clinical and ethical impact of OSA questionnaire screening in populations with high cardiovascular risk such as patients with type 2 diabetes. The overarching goal of this article is to advocate OSA screening in type 2 diabetes patients using simple home sleep monitoring devices instead of inaccurate and therefore potentially dangerous questionnaires.

Screening questionnaires were introduced to improve the recognition of patients suffering from moderate or severe OSA (indicated for treatment), particularly in primary care practice, where such patients remained largely unrecognized without systematic screening. The various questionnaires deployed for screening summarize and quantify the presence of risk factors that are strongly associated with OSA – namely, obesity, large neck circumference, hypertension, snoring, and different symptoms of daytime tiredness/sleepiness. However, it has proved to be a considerable challenge to create a questionnaire that would be both (1) sensitive enough to minimize the chance of marking patients falsely as being at low risk of OSA and therefore inadvertently denying them appropriate treatment and (2) specific enough to minimize the chance of inappropriately marking healthy patients as being at high risk of OSA and leading them to the unnecessary use of medical resources.

One of the first questionnaires to be developed and which remains widely used is the Berlin questionnaire, an outcome of the 1966 Berlin Conference on Sleep in Primary Care. The usefulness of the Berlin questionnaire as a tool to recognize patients with OSA was consequently validated in a study comparing the performance of the Berlin questionnaire with polysomnography (which is the gold standard for the diagnosis of OSA). The sensitivity and specificity of the Berlin questionnaire to identify moderate or severe OSA (i.e., a level of severity where treatment is recommended) were found to be 0.54 and 0.97, respectively (14). Soon after publication, these results were challenged by other clinicians, who recalculated the published data and found a sensitivity of 0.95 and specificity of 0.48 (15). Nevertheless, the major limitation of the study resides in the fact that the high-risk subjects identified by the Berlin questionnaire were over-represented in the polysomnography subgroup (69% high-risk subjects, while there were only 38% high-risk subjects in the whole study population). The limited number of low-risk subjects as identified by the Berlin questionnaire that were recruited into the polysomnography group (31 subjects) also resulted in a low number (exactly one patient) of subjects with polysomnographically verified severe OSA and nobody with moderate OSA. Clearly, the insufficient sample size of the study makes sensitivity and specificity calculations unreliable. Consequently, several studies retested the performance of the Berlin questionnaire in various countries and in different patient populations (primary care patients, preoperative patients, patients referred for suspected OSA, or sleep clinic patients), and they reported conflicting results with sensitivity and specificity ranging from 0.43 to 0.89 and 0.33 to 0.79, respectively (16–19). In summary, the Berlin questionnaire improves the identification of patients with OSA; however, its performance seems to be variable and uncertain in studied populations.

In 2006, the STOP and its upgraded version, the STOP Bang questionnaires, were introduced and applied in preoperative patients (20). Both questionnaires score one point for the presence of some of the symptoms such as snoring, tiredness, observed pauses in breathing, high blood pressure, BMI, age, neck circumference, and gender. The initial performance characteristics of the STOP Bang questionnaire validated on 177 preoperative patients were promising, finding sensitivity 0.92, and specificity 0.43 to detect moderate or severe OSA (using a total questionnaire scoring of ≥5). With continued recruitment and an increased number of assessed subjects to 746, sensitivity dropped to 0.23 and specificity increased to 0.56. In an effort to increase the questionnaire sensitivity, it was suggested to lower the score threshold indicative of being at high risk of having OSA to 3 points, which helped to increase sensitivity to 0.68 but at the cost of specificity, dropping to 0.11 (21). Furthermore, no study to date evaluated the questionnaire separately for men and women even though gender plays a major role in the outcome of scoring. The key advantage of the STOP and STOP Bang questionnaires is their straightforward application and interpretation. However, their performance characteristics are too limited to justify their use as a screening tool for such a serious condition in a population that is already affected by a high incidence of cardiovascular events.

A number of other questionnaires were also developed and used to screen subjects for OSA, e.g., Epworth’s Sleepiness Scale, the ASA Checklist, the Sleep Apnea of Sleep Disorders Questionnaire, the Wisconsin Questionnaire, or the Four-Variable Screening Tool. Although some of them performed better than others, none of them was convincing enough to become widely adopted in practice. The inherent limitation of any OSA screening questionnaire is that some of the answers are often based on the patient’s judgment and self-perception. For example, when asked about snoring, patients typically rely on the information from their bed partners. However, patients sleeping in separate bedrooms or living alone might not be able to answer appropriately. Similarly, tiredness/sleepiness is frequently perceived as a natural symptom of aging or attributed to other chronic diseases.

It should be emphasized that a false negative result from an OSA screening questionnaire might have serious clinical, ethical, and forensic connotations for a type 2 diabetes patient (therefore in elevated cardiovascular risk) who is suffering from moderate or severe OSA but who is deprived of therapy if inappropriately marked as being at low risk. In contrast to questionnaires, technological advancements in recent years have opened new possibilities for accurate OSA screening in the form of various home sleep monitoring devices (typically recording breathing and hemoglobin saturation). These devices were shown to have an acceptably high sensitivity and specificity ~0.90 (22–24). The overall complexity of using home sleep monitoring devices is similar to a routinely performed 24-h ECG or a blood pressure monitoring device. The growing availability and affordability of home sleep monitoring devices raises the fundamental question if it is sensible and clinically acceptable to use questionnaires as the first screening step. In answer to this, clinicians should examine the evidence keeping in mind the best interests of the patient. Our analysis found that due to the low sensitivity of questionnaires, questionnaire-based OSA screening fails to identify up to 57% of patients with moderate or severe OSA (who should receive treatment) while reassuring them that they are at low risk of OSA. Support for proceeding directly to OSA screening using only home sleep monitoring without prior stratification by questionnaire into high- and low-risk groups is strengthened by the fact that a substantial number of subjects (in our experience about 50% of patients with type 2 diabetes) are identified as high risk by questionnaires and anyway subsequently undergo home sleep monitoring as a second step in the current two-step OSA screening approach.

To conclude, the vast majority of patients with OSA are not aware of any illness and they remain undiagnosed. However, OSA is an important cardiovascular risk factor highly prevalent in type 2 diabetes patients. Between 25 and 30% of patients with type 2 diabetes who are already at high cardiovascular risk because of diabetes also suffer from moderate or severe OSA and should be treated as the treatment reduces mortality to the control group level. Therefore, physicians should improve the detection of OSA ideally *via* a systematic, precise, and reliable screening program. In our view, the recommended optimal OSA screening procedure should be evidence-based and reflect technological advancements in the field of sleep medicine. Questionnaire-based screening for OSA in the type 2 diabetes population is not supported by scientific evidence and might have a consequence as discussed above. Therefore, we advocate using home sleep monitoring devices as the first and only screening tool in all patients with type 2 diabetes as they provide an objective OSA assessment with proven high sensitivity and specificity to a level that approaches the gold standard polysomnography diagnosis. Moreover, they are easy to use by both patients and medical care professionals. Nevertheless, screening questionnaires still represent a useful tool for OSA screening in the general population where the prevalence of OSA is much lower compared to type 2 diabetes population and where the clinical consequences of a false negative finding are not so detrimental due to the overall lower cardiovascular risk profile.

## Author Contributions

JP and KW participated in the data collection and manuscript preparation.

## Conflict of Interest Statement

The authors declare that the research was conducted in the absence of any commercial or financial relationships that could be construed as a potential conflict of interest.
